# The effects of Teen Clubs on retention in HIV care among adolescents in Windhoek, Namibia

**DOI:** 10.4102/sajhivmed.v21i1.1031

**Published:** 2020-02-03

**Authors:** Farai K. Munyayi, Brian van Wyk

**Affiliations:** 1 School of Public Health, Faculty of Community and Health Sciences, University of the Western Cape, Cape Town, South Africa; 2 International Training and Education Center for Health, Windhoek, Namibia

**Keywords:** adolescents, HIV, retention in care, Teen Club, antiretroviral therapy, lost to follow-up, group interventions, adolescent-friendly HIV services

## Abstract

**Background:**

Adolescents living with HIV (ALHIV) are notably underserved by the national HIV programmes globally because of their unique needs. Of particular concern is limited access to and availability of adolescent-friendly antiretroviral therapy (ART) services, which contribute to poor retention in care in many sub-Saharan African countries. A Teen Club intervention was introduced in 2010 in Windhoek, Namibia, to improve retention in care among ALHIV through psychosocial support in a peer-group environment.

**Objectives:**

To compare the effects of the Teen Club intervention against standard care on retention in HIV care amongst adolescents at a Paediatric ART clinic.

**Method:**

A retrospective cohort analysis of adolescents aged 10–19 years receiving ART between July 2015 and June 2017 was conducted. Routine patient data were extracted from an electronic database and patient registers. A sample of 385 participants was analysed: 78 in the Teen Club and 307 in standard care. Retention was measured by assessing attendance to prescribed clinic visits up to 24 months. Comparisons were assessed with the Chi-square test, and Kaplan–Meier survival analysis was conducted to analyse differences in retention rates.

**Results:**

The overall retention rate at 24 months among all adolescents was 90.1%, with no statistically significant difference between those in Teen Club (91%) and those in standard care (89%) (*p* = 0.956). Younger adolescents (10–14 years) had better retention rates at 24 months compared to older adolescents (15–19 years) (94% vs. 86%; *p* = 0.016). Retention rates were significantly higher for adolescents on first-line ART regimen (vs. second line: hazard ratio [HR] = 0.333; 95% confidence interval [CI] = 0.125–0.889); on ART ≥ 12 months (vs. < 12 months: HR =0.988; 95% CI = 0.977–0.999); and those to whom their HIV status was disclosed (HR = 0.131; 95% CI = 0.025–0.686).

**Conclusion:**

Group-based adherence support interventions did not improve retention rates for younger adolescents in specialised paediatric ART clinics but may still hold the potential for improving retention rates of older adolescents.

## Introduction

Worldwide, in 2015, an estimated 1.8 million adolescents aged between 10 and 19 years were living with HIV, with nearly 80% of all new HIV infections among adolescent girls occurring in sub-Saharan Africa.^1^ The World Health Organization (WHO) defines adolescents as children or young adults aged between 10 and 19 years.^2^ In many sub-Saharan Africa countries, HIV services are organised around adult or paediatric care, with the paediatric care facilities being responsible for the treatment of younger adolescents living with HIV (ALHIV). These facilities are often ill-equipped to provide guidance on adolescent-specific issues for older adolescents, such as information related to their needs to deal with sexual and reproductive health (SRH) concerns.^3^ The ALHIV are often lost in the transition gap from paediatric to adult HIV programmes, resulting in poor retention in care and poor adherence to antiretroviral therapy (ART), especially in the 15–19 years age group.^4^ A study in Uganda reported that the risk of non-retention in HIV care was significantly greater in older adolescents (15–19 years) compared to the 10–14 years age group.^3^

Poor retention in HIV care is a major public health challenge, and interventions to ensure persistent engagement in care are essential for sustaining substantial public health and individual benefits.^5^ Health care providers are faced with challenges emanating from high rates of non-retention among adolescents. The 15- to 24-year age group has been described as the fulcrum of the epidemic, with 42% of new infections occurring in this age group in 2010.^6^ In 2010, a Teen Club intervention was introduced at a tertiary HIV clinic in Malawi to provide ALHIV on ART with dedicated clinic time, peer mentorship, SRH education, ART refill, and support for positive living and treatment adherence. An evaluation of the programme in 2015 found that ALHIV with no Teen Club exposure were less likely to be retained than those with Teen Club exposure (adjusted odds ratio [aOR] 0.27; 95% CI 0.16, 0.45). Adolescents living with HIV aged 15–19 years were more likely to have attrition from care than those aged 10–14 years (aOR 2.14; 95% CI 1.12, 4.11).^7^ Concerns have been raised about waning adherence over time, including loss of patients from HIV programmes when scaling up.^8^ Recommendations for monitoring long-term retention rates, and the development of evidence-based interventions to address problems, especially among adolescents, have been put forward.^8^

Namibia has adopted the fast track goals of the Joint United Nations Programme on HIV and AIDS to control the HIV epidemic by 2030. The fast track goals are aimed at ensuring that 90% of people living with HIV (PLHIV) are identified; 90% of those identified are effectively linked and retained on ART and that 90% of these achieve viral suppression.^9^ In Namibia, 15- to 25-year olds reportedly have only 64.5% (females) and 60.7% (males) viral suppression; which is below the national average suppression levels for older adults on ART at 92.5% and 86.3% for females and males respectively.^10^

The HIV programmes of several countries have demonstrated the effectiveness of group-based interventions in improving retention in care among adults. A systematic review of literature on improving retention among adolescents and adults in low- and middle-income countries reported that only one study evaluated HIV programmes for youth (15–24 years) while several studies reported associations between task shifting of services, down-referral of stable patients, decentralisation, differentiated care and retention in care rates among adults.^11^ A study of community-based service delivery that was effective for adults was found to be not effective among the youth.^11^ An evaluation study on the effect of a novel adolescent and youth Red Carpet Programme on linkage to care and outcomes conducted in health care facilities and schools in Homa Bay County, Kenya, showed that when compared to the pre-implementation cohort, retention on ART for the post-implementation cohort increased from 66% to 90% at 3 months, and from 54.4% to 98.6% at 6 months.^12^ The WHO proposes support groups as an intervention for improving retention in care for persons receiving ART, and that the effectiveness can be maximised if support groups are formed around homogenous population groups such as couples, men having sex with men or adolescents.^13^

Adolescent transition to adult care outcomes have been reported mostly in the Global North, but these types of evaluations are less available in sub-Saharan Africa because of limited resources that increase the complexity of transition and its assessment.^14^ Multicountry assessments conducted in Africa reported some of the transition challenges as a lack of appropriately trained staff; a lack of specialist adolescent-friendly services, policies, guidelines and training; and a lack of health education especially to address SRH and planning.^15^ A study in South Africa reported adolescent and health care provider resistance to transition because of the strong attachments and relationships built over time, as well as a lack of communication between paediatric and adult care providers.^16^ In Namibia, adolescent transition involves achievement of three goals: (1) Full disclosure by the age of 10–12 years, with an understanding of HIV prevention measures and linkage to an adolescent support group; (2) understanding of medication by those aged 13–16 years, including adherence/appointment keeping and being part of a support group; and (3) understanding of the importance of medication adherence in the last two to three clinic visits by those aged 17–19 years, with encouragement for the adolescent to attend clinic visits independently, and if the adolescent is 19 years and has achieved goal 3, transitioning them to adult care with their consent. Paediatric care staff follow up the transitioned adolescent for at least one suppressed viral load.^17^

A Teen Club intervention was established in 2010 at the Intermediate Hospital Katutura Paediatric ART Clinic in Windhoek to address the unique needs of adolescents on HIV treatment.^18^ The Teen Club aims to improve retention in HIV care through, among other activities, psychosocial support, HIV counselling and health education. The Teen Club is a psychosocial peer support group and does not offer special arrangements for clinic or treatment (pill pickup) visits. All adolescents receive their medication through an appointment system, with a standard treatment and clinical monitoring schedule. Although the Teen Club is a voluntary peer support group, the clinic staff encourage all adolescents who have completed their HIV disclosure process to enrol in the Teen Club (see comparison of services offered in Standard care vs Teen Club in Table 1). To date, the effectiveness of the Teen Clubs on retention of adolescents in care has not been formally evaluated in Namibia. This article reports on the effects of the Teen Club intervention against standard care on retention in care among adolescents at the clinic.

**TABLE 1:** Comparison of standard care and Teen Club care.

Standard care	Teen Club
Adolescents should have full disclosure by the age of 10–12 years; disclosure can be delayed depending on the cognitive ability of the adolescent	Adolescents should have full disclosure; this is a prerequisite for enrolment into the Teen Club
Goal-related transition from paediatric/adolescent to adult HIV services	Goal-related transition from paediatric/adolescent to adult HIV services
Routine viral load monitoring and targeted viral load monitoring for suspected treatment failure	Routine viral load monitoring and targeted viral load monitoring for suspected treatment failure
Age-appropriate and developmentally appropriate adherence counselling	Age-appropriate and developmentally appropriate adherence counselling
Lost to follow-up/defaulter tracking and tracing	Lost to follow-up/defaulter tracking and tracing
HIV treatment literacy training of guardians and caregivers on treatment adherence, disclosure and stigma issues	HIV treatment literacy training of guardians and caregivers on treatment adherence, disclosure and stigma issues
Routine discussion with the children on their experience at school and future plans	Routine discussion with the child on their experience at school and future plans
Linkage to relevant stakeholders and social support mechanisms in the community	Linkage to relevant stakeholders and social support mechanisms in the community
Age-appropriate psychosocial support includes individualised and group counselling on issues such as treatment failure counselling, opportunistic infections, STIs, SRH, alcohol use and abuse, mental health, child protection and other topics according to the adolescents’ needs.	In addition to age-appropriate psychosocial support offered in standard care, the Teen Club: ■ Meets once a month on a Friday or Saturday in ‘safe spaces’ at the clinic ■ Shares challenges, fears, experiences and coping mechanisms during monthly meetings ■ Has special talks or presentation of ALHIV-related topics from subject matter experts ■ Has access to information, education and communication materials, such as videos and dramas/acts on adolescence and HIV, followed by discussions ■ Occasionally participates in Teen Club retreats and trips where recreational activities and life stories are shared

ALHIV, adolescents living with HIV; SRH, sexual and reproductive health; STIs, sexually transmitted infections.

## Methods

### Study setting, design and population

A retrospective cohort study was conducted using medical records of HIV-positive adolescents aged between 10 and 19 years receiving ART at a hospital-based paediatric clinic in Windhoek, Namibia. The study population was stratified into two groups: adolescents attending the Teen Club and adolescents receiving standard care. Routine clinical records of the study population from 01 July 2015 to 30 June 2017 were reviewed. All adolescents aged between 10 and 19 years that attended the clinic between 1 July 2015 and 30 June 2017 as their initial enrolment site were eligible for inclusion in the study. According to the 2014 Namibian National ART Guidelines, the HIV disclosure process of the child must be a carefully planned process that informs the child of their HIV status and why they have to take their HIV medication. The process must take into account the individual’s maturity, understanding of HIV and their social support system, and should be initiated as early as 6–10 years of age.^19^ Once adolescents are aware that they are HIV infected, they can enrol in the Teen Club.

### Participants’ selection

**FIGURE 1:**
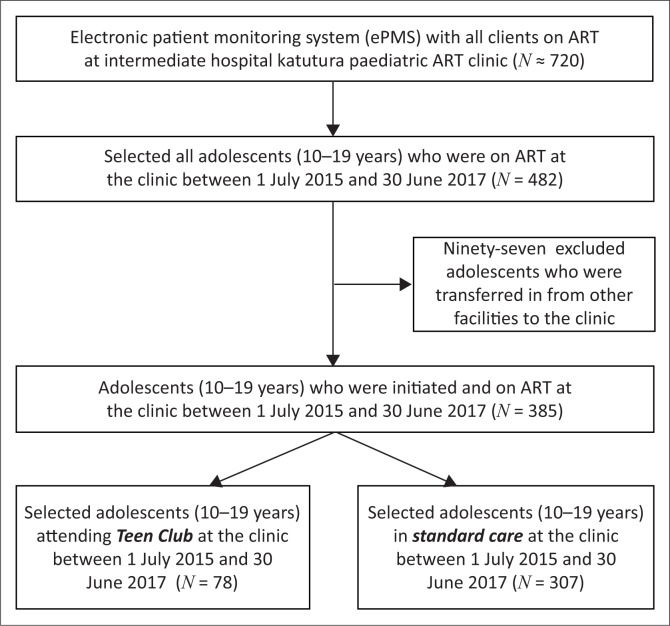
Flow chart of the sampling process for the study.

The study sample was all inclusive of the study population. Figure 1 shows that 720 children and adolescents were receiving ART at the clinic and that 482 were aged between 10 and 19 years. Eighty five of these were members of the Teen Club. Any adolescent who attended at least one Teen Club meeting was considered ‘exposed’ to the intervention. The calculated minimum total sample size using Epi Info included 272 participants, with 46 from the Teen Club stratum (exposed) and 226 in the standard care/non-Teen Club stratum (unexposed). Parameters used to calculate sample size include a power of 80%, an assumed difference of 20% between the two groups, with a 95% confidence interval, and an unexposed/exposed ratio of 5.

Patient demographics and visit details are completed routinely by health care workers and entered into individual patient care booklets (PCBs) during clinic visits. Patient information is then entered into an electronic patient monitoring system (*e*PMS) by data clerks. Patient data were extracted from the electronic database into an Excel spreadsheet. Teen Club members sign in at every Teen Club meeting. The Teen Club attendance register was reviewed to match adolescents on the *e*PMS and the Teen Club members using the unique ART numbers allocated as unique patient identifiers. Patient care booklets for adolescents with incomplete records in *e*PMS were retrieved, and the missing information was added onto the Excel spreadsheet. Extracted data were coded using R statistical package and transferred and saved onto a password-protected Excel file to ensure that the data cannot be altered. Data cleaning and preparation or coding were performed on the Excel file that was then exported into an SPSS file. Further coding and labelling of variable categories allowed conversion and compatibility with R.

### Variables

We defined retention in HIV care as being in care at 24 months, that is, the end of the study period, with evidence of clinic attendance during the study period. Participants not in care at 24 months were defined as lost: missed > 30 days, lost to follow-up (LTFU): missed > 90 days and transferred out or died. Adolescents who miss appointments are listed and followed up telephonically and, in the community, if possible, by clinic staff or defaulter tracers based at the clinic. If they cannot be tracked and brought back into care within 90 days, they are documented as LTFU. Information pertaining to clinic visits was extracted from the PCBs for participants with missing retention codes on the *e*PMS. The most important exposure variable assessed is the model of care, with Teen Club members in the exposed group and adolescents in standard care in the unexposed group. Other predictor variables included demographic and clinical characteristics, such as age, sex, period on ART, current ART regimen and HIV disclosure status. Although there are other variables that could have been of interest, for example, socioeconomic status (such as type of residence, school/work and caregiver support), this information was not available in the routine clinical records of the study participants.

### Statistical analyses

Data analysis included univariate analyses to describe demographic variables such as sex and age distribution; clinical variables such as model of care, disclosure status, period on ART and type of regimen; and retention as measured by attendance of clinic appointments at 24 months. Bivariate analysis was performed using the Chi-square test to determine the significance of associations between retention in care and selected demographic and clinical variables. The cut-off for significance was set at *p* < 0.05. If the sample size was very small in any cell (< 5), Fisher’s exact test was used as an alternative to the Chi-square test.

Risk ratios were also calculated for the comparison of retention rates between Teen Club members and adolescents in standard care. The Kaplan–Meier survival analysis with the log-rank test was used to compare the survival curves in the Teen Club and adolescents in standard care groups, and younger and older adolescents’ groups. Multivariate analyses were performed using the Cox regression method (Cox proportional hazard regression model) to analyse differences in retention rates between Teen Club members and the adolescents in standard care and other variables of interest. Time to event was defined as time to defaulting/lost, LTFU and transferred out or died. The datasets in both Excel and SPSS formats were exported to R for data analysis, and the analysis was performed using both R and SPSS statistical packages.

### Ethical considerations

This study was approved by the Namibian Biomedical Research Ethics Committee and Research Management Committee based at the Ministry of Health and Social Services, and the University of the Western Cape Biomedical Research Ethics Committee (Ethical Clearance Number: BM17/8/14 and 18/3/3FM).

## Results

A total of 482 adolescents aged between 10 and 19 years attended the paediatric ART clinic during the 2-year study period. All records of the adolescents were extracted from the *e*PMS for the 2-year period. Records of adolescents who were transferred in from other facilities, who had incorrectly entered demographic information and those with missing files/PCBs were excluded from the final study sample. A total of 385 adolescents were eligible, 78 of them being in the Teen Club. The average age among adolescents included in the study was 14 years: 51% were aged 10–14 years, and 49% were aged 15–19 years. However, Table 2 shows that the proportion of older adolescents in the Teen Club was twice as much as that of the younger adolescents (66.7% vs. 33.3%; *p* = 0.015). Most club members were female adolescents (59% vs. 41%; *p* = 0.001). Overall, 20% of the adolescents included in the study were Teen Club members while 80% were those receiving standard care. A total of 16 Teen Club meetings were held during the study period, with a mean attendance of approximately five meetings per adolescent, a median of 3 and a range of 1–14 attendances among all Teen Club members.

**TABLE 2:** The demographic and clinical characteristics of adolescent participants on antiretroviral therapy at the Intermediate Hospital Katutura Paediatric Antiretroviral Therapy Clinic (*N* = 385).

Characteristic	Standard care		Teen Club		Total	*p*
	*n*	%	*n*	%		
**Sex**						
Male	173	56.4	32	41.0	205	0.015*
Female	134	43.6	46	59.0	180	-
**Age group**						
10–14 years	171	55.7	26	33.3	197	0.001**
15–19 years	136	44.3	52	66.7	188	-
**Disclosure status (*n* = 372 )**						
Disclosed	278	94.2	77	100	355	0.031*
Not disclosed	17	5.8	0	0	17	-
**ART regimen**						
First-line regimen	226	73.6	53	67.9	279	0.318
Second-line regimen	81	26.4	25	32.1	106	-
**Duration on ART**						
< 12 months	3	1.0	0	0	3	0.382
≥ 12 months	304	99.0	78	100	382	-
**Retention in care at 24 months**						
In care	276	89.9	71	91.0	347	0.931
Lost to follow-up	18	5.9	4	5.1	22	-
Transferred out	13	4.2	3	3.9	16	-

ART, antiretroviral therapy.

* ,Correlation is significant at 0.05 level (2-tailed).

** ,Correlation is significant at 0.01 level (2-tailed).

All the adolescents in the Teen Club had their HIV status disclosed to them (a requirement for enrolment in the Teen Club), compared to 94% of adolescents in standard care (*p* = 0.031). The median duration on ART among all participating adolescents was 10.3 years (interquartile range IQR = 7.7–11.7). There was no significant difference between Teen Club members and those in standard care in duration on ART (100% vs. 99% for 12 months or more), type of regimen (68% vs. 74% on first line) and retention in care at 24 months (91% vs. 90%).

**FIGURE 2:**
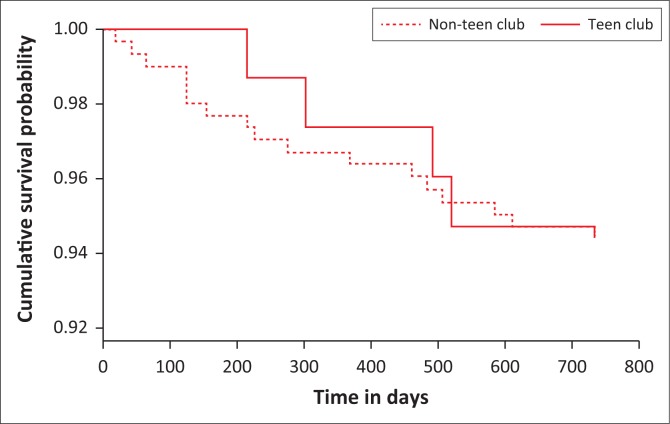
Kaplan–Meier survival analysis for retention in care among Teen Club members and adolescents in standard care over time.

Figure 2 shows that the probability of being retained in care in the first 500 days (16 months) among Teen Club members was higher when compared to adolescents in standard care. In sensitivity analysis, there were no statistically significant differences in retention in care at 24 months between males and females (*p* = 0.343), between adolescents disclosed to and adolescents not disclosed to (*p* = 0.343), between adolescents on first-line ART regimen and those on second-line regimen (*p* = 0.269) and between adolescents on treatment < 12 months and those who had been on treatment for ≥ 12 months (*p* = 0.847).

**FIGURE 3:**
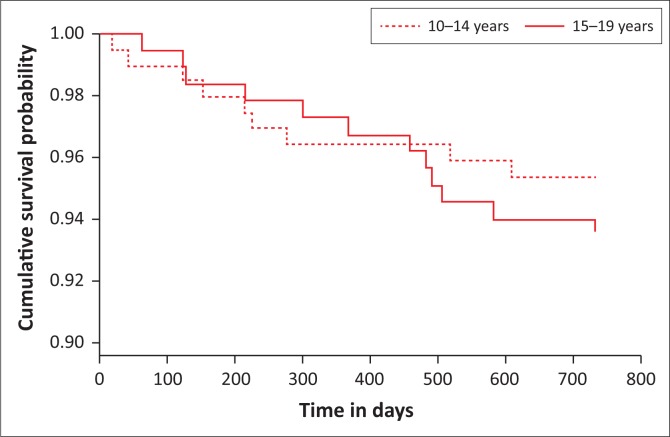
Kaplan–Meier survival analysis for retention in care among younger and older adolescents over time.

Figure 3 shows that the probability of being retained in care is similar between younger and older adolescents for approximately the first 500 days (16–17 months). Then, the likelihood of being retained in care was higher among younger adolescents when compared to older adolescents.

The log-rank test indicated no statistically significant difference in the survival curves between younger and older adolescents during the 2-year period (*p* = 0.4). However, there was a statistically significant difference in retention in care at 24 months between younger and older adolescents (*p* = 0.016). The chance of being retained in care among older adolescents at 24 months was less compared to younger adolescents (risk ratio [RR] = 0.918; 95% CI = 0.858–0.982).

Table 3 indicates that the hazard ratio for model of care was 0.607; that is, the hazard rate for being lost from care among Teen Club members was approximately 40% less compared to adolescents in standard care. Nonetheless, no statistically significant difference in hazard rates was confirmed between the two groups (*p* = 0.456). The hazard ratio per age group was 1.305; that is, the hazard rate for being lost from care among the older adolescents was approximately 30% higher when compared to the younger adolescents. Retention rates were higher among younger adolescents. This was significant at the 1% level (*p* = 0.008).

**TABLE 3:** Cox proportional hazard regression model.

coef	coef	exp(coef)	se(coef)	*z*	Pr(>|*z*|)
Care	−0.498589	0.607387	0.669281	−0.745	0.45629
Age	0.266007	1.304744	0.100969	2.635	0.00843 **
Sex	−0.467084	0.626828	0.507478	−0.920	0.35736
Disclosure	−2.031364	0.131157	0.844418	−2.406	0.01614 *
Regimen	−1.099095	0.333172	0.500542	−2.196	0.02811 *
Duration	−0.012241	0.987834	0.005798	−2.111	0.03476 *

Note: See Cox Proportional Hazard Regression model calculations in Appendix 1.

Significance codes: ‘**’ 0.01 ‘*’ 0.05 ‘.’ 0.1 ‘ ’ 1.

Concordance = 0.755 (se = 0.07).

R-square = 0.044 (max possible = 0.416).

Likelihood ratio test = 16.63 on 6 df, *p* = 0.01.

Wald test = 16.47 on 6 df, *p* = 0.01.

Score (log-rank) test = 17.99 on 6 df, *p* = 0.006.

The hazard rate for being lost from care among adolescents on a first-line ART regimen was approximately 67% less compared to those on the second-line regimen, with a statistically significant difference at the 5% level (*p* = 0.028). Hazard rates for duration on ART (HR = 0.988; *p* = 0.035) and disclosure status (HR = 0.131; *p* = 0.016) were also significant, and indicated that adolescents on ART for ≥ 12 months had less chance of being LTFU than those on ART < 12 months at the beginning of the study, and that ‘disclosed’ adolescents had less likelihood of being LTFU compared to those not disclosed.

## Discussion

This study found the overall retention in care rates to be approximately 90.1% at 24 months among all adolescents included in the study. Similar retention rates have been observed in other studies conducted in South Africa (89% and 83%).^20^ A study in Uganda reported that 90% of adolescents were retained in care at 12 months after ART initiation^21^ while another study reported rates of 96%, 90% and 83% at 6, 12 and 24 months, respectively.^3^

Although the study produced similar retention rates between adolescents in the Teen Club and those in standard care at 24 months, there were better retention rates among the Teen Club members. Comparable rates were reported in South Africa, although the authors reported bigger differences of 95% retention among adolescents attending a dedicated adolescent clinic as compared to 85% (odds ratio [OR] = 3.7; 95% CI 1.2, 11.1; *p* = 0.018) for adolescents in standard care.^20^ A study in Kenya observed no difference in retention rates at 6 and 12 months between adolescents accessing youth-friendly services and those attending selected facilities prior to the implementation of youth-friendly services.^22^ Decreasing retention rates were also observed in our study in both adolescents in the Teen Club and those in standard care. Decreases with time from high initial retention rates among adolescents have been previously documented in other studies in Southern Africa.^20^

Notably, there were significant differences observed in this study in retention rates between younger and older adolescents at 24 months, with younger adolescents having better retention rates. This finding is supported by observations reported in other studies conducted in other low-and middle-income countries where the risk of non-retention among older adolescents was 1.30 times higher than the risk in younger adolescents at 24 months (95% CI 1.02–1.66).^3^ Lower hazard rates among younger adolescents could be attributed to paediatric clinics being designed to care better for younger adolescents than older ones, and younger adolescents still benefiting from caregiver support.^23^ Higher rates of attrition among older adolescents may be because of challenges associated with treatment fatigue or transition from paediatric to adult care and adulthood. The fact that the Teen Club had twice as many older adolescents as younger adolescents may have negatively influenced the comparative magnitude of the difference in retention rates between club members and adolescents in standard care. The Malawi Teen Club evaluation reported similar results, and recommended that prospective evaluations of the Teen Club package with higher methodological quality should be conducted for programmes in low- and middle-income settings to prioritise interventions for ALHIV.^7^

The study also showed that adolescents who had been disclosed to were less likely to be LTFU. There was enough evidence in support of reduced hazard rates among adolescents who had their HIV status disclosed to them as compared to those who had not gone through the disclosure process. Disclosure of HIV status is an essential component of paediatric HIV care and long-term HIV management, and an important step towards the transition from paediatric into adolescent and eventually adult care.^24^ A study at a county referral hospital in Kenya found a direct correlation between disclosure and retention in care, with overall retention at 64% for adolescents not disclosed to, 82% for partial disclosure and 92% among adolescents who had full disclosure.^25^ Failure to disclose HIV status may result in poor understanding of why an adolescent needs to stay in care, which may negatively influence retention rates among adolescents. The overall high rate of disclosure among all adolescents in our study may have contributed to the relatively high overall retention rate, and in particular better retention rates among adolescents in the Teen Club in which 100% of the adolescents were disclosed to.

Studies have shown that adolescent-friendly HIV services can lead to substantial improvements in treatment outcomes among adolescents, which can be comparable to better treatment outcomes among children and adults. Furthermore, retention in care support through peer support groups is reportedly an effective strategy to improve treatment outcomes for individuals on ART in general. The Intermediate Hospital Katutura Paediatric ART Clinic provides specialised HIV services for all children and adolescents (those receiving standard care and members of the Teen Club), with dedicated staff, systems and a conducive environment to address children- and adolescent-specific treatment issues. In addition, the clinic introduced the Teen Club as an intervention beyond standard care to further enhance retention rates among adolescents through peer support, health education and psychosocial support. Generally, the clinic prescribes shorter clinic visit intervals for unstable adolescents (with high viral loads or social issues) and recommends differentiated service delivery for stable adolescents (multi-month dispensing) and referrals to psychosocial services, among other interventions, to improve retention rates among all the adolescents accessing services at the facility.

Our study found no statistically significant differences in retention rates between adolescents in the Teen Club and those in standard care, although there were slightly higher retention rates among the Teen Club members. The relatively high retention rates are comparable to rates reported in studies performed elsewhere in adolescent-friendly HIV service settings. Of note, significant differences were found between younger and older adolescents, which is in line with what has been reported elsewhere globally. These results may only apply to this study population and setting. However, the effectiveness of the Teen Club intervention can reasonably be generalised to ALHIV who are between 10 and 19 years, and accessing ART at a specialised paediatric ART clinic.

We concluded that in a specialised paediatric ART clinic, group-based interventions may not substantially improve retention rates. Targeted, individual and age-specific interventions may provide additional benefits to the Teen Club intervention.

In such settings, there is a need to strengthen individual case management strategies for adolescents with poor treatment outcomes. Although group-based interventions may improve overall retention rates among adolescents, special attention is needed for those failing treatment. Poor retention rates may be because of patient-related factors such as active substance abuse, depression, non-disclosure or not feeling well. Factors related to health care system (dissatisfaction) or medication (type of antiretrovirals, pill burden, side effects, etc.) must also be explored on an individual basis. The national programme should also consider scaling up differentiated care models that are tailored for younger and older adolescents, which may include weekend HIV services.

The effect of age in the Teen Club cannot be underestimated. We recommend provision of age-specific interventions/activities within the Teen Club intervention, with topics specifically targeting older adolescents, such as SRH, education and career concerns, and other issues on transition to adulthood. The current Teen Club model that only provides psychosocial support as an additional benefit might explain the minimal differences in treatment outcomes between the adolescents in the Teen Club and those in standard care. Modification of the Teen Club to an Adolescents Treatment Adherence Club with special arrangements for pill pickups, clinical monitoring and fast-tracking of consultations with health care workers may provide additional benefits and improve uptake of the intervention. Overall, having a specialised paediatric HIV clinic demonstrated that improved treatment outcomes can be achieved among adolescents, which is imperative to close the gap towards achieving the 90–90–90 goals. National programmes must consider decentralising paediatric and adolescent-friendly HIV services, and individual-based interventions could substantially improve retention rates among adolescents, and close the gap between this underserved group and that of children and adults.

## Limitations of the study

The use of a retrospective cohort study design meant that we had to rely on the accuracy of the record-keeping by the health care workers. Reliance on routinely collected data meant that we could not control the exposures and outcomes of interest. The level of exposure to the intervention could have also affected the results, as those adolescents who attended at least one Teen Club meeting were considered to have been exposed to the intervention. The inclusion of adolescents transferred out in the non-retention group may have affected the results; however, the numbers were small and were not significantly different between the two groups. The amount of missing data and files could have potentially influenced the findings of the study. However, it was predominantly some viral load results that were missing on the *e*PMS but filed in the PCBs, and 13 (3.4%) of the adolescents did not have a disclosure status recorded. The inequivalence in sample sizes between the comparison groups (Teen Club Members vs. adolescents in standard care) could also potentially result in a type II error.

## Conclusions

Worldwide, adolescents on ART are reportedly at higher risk of non-retention in HIV care, compared to children and adults. Studies have shown that adolescent-friendly HIV services can lead to substantial improvements in treatment outcomes among adolescents on ART, which can be comparable to treatment outcomes among children and adults. Peer support groups are reportedly an effective strategy to improve treatment outcomes for individuals on ART in general. This study sought to compare the retention in care rates between adolescents attending the Teen Club and those receiving standard care during the period 1 July 2015 to 30 June 2017.

Our study found no significant differences in retention rates between adolescents that were in the Teen Club and those in standard care, although there were slightly higher retention rates among Teen Club members. Overall, retention rates were at 90.1% at 24 months among all adolescents included in the study, and these relatively high retention rates are comparable to rates reported in other studies that assessed treatment outcomes in adolescents receiving treatment in adolescent-friendly HIV services.

In addition, our study also sought to establish any associations between retention rates and other demographic and clinical characteristics of the adolescents receiving treatment at the clinic. Of note, significant differences were found between younger and older adolescents, which is in line with what has been reported elsewhere globally that younger adolescents have better retention rates than older adolescents. The results also showed that HIV disclosure is a key factor in improving retention rates among adolescents on ART. These results may only apply to this particular study population and setting. However, effectiveness of the intervention can reasonably be generalised to ALHIV who are accessing ART at a specialised adolescent-friendly ART clinic. We concluded that in a specialised paediatric ART clinic, group-based interventions may not substantially improve retention rates among adolescents on ART. Targeted, individual and age-specific interventions may provide additional benefits to the Teen Club intervention, particularly among older adolescents. Modification of this Teen Club model to include dedicated clinic time, peer mentorship, ART refill and additional treatment adherence support may also improve overall treatment outcomes. The results provide preliminary data that can be a vehicle for further research, modification of the intervention and development of future prospective studies.

## Appendix 1

### The Cox proportional hazard regression model

The fitted Cox proportional hazard regression model was:

$$\log\left(\frac{h(t)}{h_{0}(t)}\right)=-0.499X_{1}+0.266X_{2}-0.467X_{3}-2.031X_{4}-1.099X_{5}-0.012X_{6}$$

Or equivalently,

$$\begin{array}{l} \frac{h(t)}{h_{0}(t)}=\exp\left(-0.499X_{1}+0.266X_{2}-0.467X_{3}-2.031X_{4}-1.099X_{5}-0.012X_{6}\right)\\ \;=0.607X_{1}+1.305X_{2}+0.627X_{3}+0.131X_{4}+0.333X_{5}+0.988X_{6} \end{array}$$

where

*h*(*t*) = expected hazard of retention at time *t*

*h_0_*(*t*) = baseline hazard of retention at time *t*

*X_1_* = model of care

*X_2_* = age group

*X_3_* = sex

*X_4_* = disclosure

*X_5_* = regimen

*X_6_* = duration on ART.
